# Evaluating the utility of effective breeding size estimates for monitoring sea lamprey spawning abundance

**DOI:** 10.1002/ece3.10519

**Published:** 2023-09-20

**Authors:** Ellen M. Weise, Kim T. Scribner, Olivia Boeberitz, Gale Bravener, Nicholas S. Johnson, John D. Robinson

**Affiliations:** ^1^ Department of Fisheries and Wildlife Michigan State University East Lansing Michigan USA; ^2^ Department of Integrative Biology Michigan State University East Lansing Michigan USA; ^3^ Fisheries and Oceans Canada Sea Lamprey Control Centre Sault Ste. Marie Ontario Canada; ^4^ U.S. Geological Survey Great Lakes Science Center, Hammond Bay Biological Station Millersburg Michigan USA; ^5^ Present address: Department of Biology Dalhousie University Halifax Nova Scotia Canada; ^6^ Present address: Pacific States Marine Fisheries Commission Portland Oregon USA

**Keywords:** effective breeding size, environmental variables, pedigree analysis, *Petromyzon marinus*, RAD capture, sampling effects

## Abstract

Sea lamprey (*Petromyzon marinus*) is an invasive species that is a significant source of mortality for populations of valued fish species across the North American Great Lakes. Large annual control programs are needed to reduce the species' impacts; however, the number of successfully spawning adults cannot currently be accurately assessed. In this study, effective breeding size (*N*_b_) and the minimum number of spawning adults (*N*_s_) were estimated for larval cohorts from 17 tributaries across all five Great Lakes using single nucleotide polymorphisms (SNP) genotyped via RAD‐capture sequencing. Reconstructed larval pedigrees showed substantial variability in the size and number of full‐ and half‐sibling groups, *N*_b_ (<1–367), and *N*_s_ (5–545) among streams. Generalized linear models examining the effects of stream environmental characteristics and aspects of sampling regimes on *N*_b_ and *N*_s_ estimates identified sample size, the number of sampling sites, and drainage area as important factors predicting *N*_b_ and *N*_s_. Correlations between *N*_b_, *N*_s_, and capture–mark–recapture estimates of adult census size (*N*_c_) increased when streams with small sample sizes (*n* < 50) were removed. Results collectively indicate that parameters estimated from genetic data can provide valuable information on spawning adults in a river system, especially if sampling regimes are standardized and physical stream covariates are included.

## Graphical abstract

Genetic assessment of 17 spawning populations of sea lamprey found substantial variability in effective breeding size (*N*_b_) and the minimum number of spawners (*N*_s_) across streams. Additionally, *N*_b_ and *N*_s_ were found to have a correlation with adult census size estimates when sampling regimes were considered. Collectively, genomic techniques provided unique information about spawning that can be used to inform future action in an annual management framework.

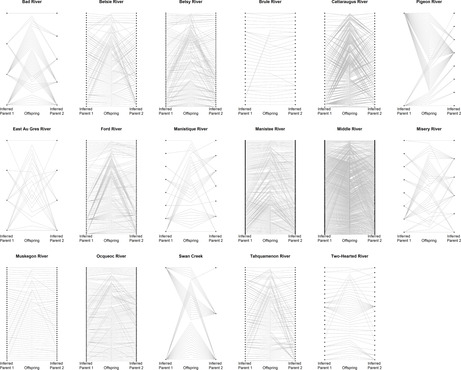

## 1 INTRODUCTION

Sea lamprey (*Petromyzon marinus*) invaded the North American Laurentian Great Lakes after the expansion of the Welland Canal in 1919. Subsequent increases in abundance were partially responsible for declines of numerous native fish species (Lawrie, 1970). To limit sea lamprey abundance in the Great Lakes, a binational control program was implemented in the mid‐20th century (Smith & Tibbles, 1980). The success of this program is evaluated using assessment techniques targeting the multi‐stage life cycle of sea lamprey, emphasizing the spawning and larval phases during accessible periods of stream occupancy, which spans several years (Applegate, 1950).

Annual surveys that quantify larval abundance, body size (a surrogate of age), and relative distribution are used to prioritize streams for treatment with the lampricide 3‐trifluoromethyl‐4‐nitrophenol (TFM; Jubar et al., 2021). Separating age classes with length alone is difficult, particularly for larger larvae (Dawson et al., 2009). While additional information about the stream environment, such as growing degree days, can improve cohort determination using length, accurate age estimation remains challenging (Dawson et al., 2020). The number of spawning adults entering streams is assessed using annual trapping and capture–mark–recapture (CMR) methods in several index streams in each Great Lake (Adams et al., 2021). CMR is an effective technique for estimating the total number of adults that could spawn in the stream (i.e., adult census population size; *N*_c_). However, the sampling framework cannot be implemented in a large number of streams due to both environmental conditions that complicate assessment and the high costs associated with evaluating a larger number of streams (Robinson et al., 2021). Furthermore, the assumptions associated with mark–recapture adult abundance estimates have not been fully tested.

Violation of assumptions of CMR models, including behaviors affecting probability of capture, trap efficiency, and low recapture rates, can complicate adult abundance estimates in systems where CMR is conducted (Bravener & McLaughlin, 2013). Additionally, trapping efforts provide information on lamprey entering streams, rather than the number of spawning individuals in sampled tributaries. Environmental variables like stream drainage area, amount of larval habitat, and the number of years since TFM treatment can affect census size estimates of spawning sea lamprey (Mullett et al., 2003). Complementary data to spawning‐ and larval‐phase sea lamprey assessment, including genetic estimates of the effective and minimum number of breeding individuals (*N*_b_ and *N*_s_, respectively) as surrogate measures of adult abundance, could improve the quantification of this control performance measure, especially in locations where trapping is difficult or intensive monitoring is not possible.

Genomic data and reconstructed pedigrees can provide population‐level inference regarding estimates of the minimum number of spawning adults (*N*_s_) and the effective number of breeding adults (*N*_b_), that can be used to fill information gaps in the absence of information on *N*_c_ and inform management actions for invasive species (Weise et al., 2020). Effective population size (*N*_*e*_) is a parameter describing the size of an idealized population that experiences drift or inbreeding at the same rate as the sampled population (Wright, 1931). *N*_*e*_ is generally calculated on a generational basis (Waples, 2016a; Waples et al., 2014), but the effective number of breeding adults (*N*_b_) can be estimated for individual cohorts with appropriate sampling. Notably, the accidental inclusion of multiple cohorts can bias *N*_b_ estimates (Robinson & Moyer, 2013; Wang, 2009; Waples, 2005; Waples & Antao, 2014). *N*_b_ estimation can be complicated by skewed sex ratios, large census size, and highly dispersed species distribution and sampling regimes (Waples et al., 2018). Since *N*_b_ is estimated for a single reproductive event, it is used to assess species for early detection of population decline or extinction risk (Jay et al., 2014; Kamath et al., 2015). For invasive species, *N*_b_ can be used to track the growth and spread of invasive species, or evaluate management intervention (Weise et al., 2020). Several methods are used to estimate *N*_b_. The linkage disequilibrium (Waples & Do, 2010) method uses non‐random associations between alleles in a set of loci to estimate *N*_b_. LD can be caused by physical linkage through proximity in the genome and by finite breeding population size (Hill, 1981, p. 19). Correlations in allele frequencies between loci that are not physically linked thus provide an estimate of *N*_b_. In contrast, the sibship frequency (SF; Wang, 2009) method uses reconstructed pedigrees to estimate *N*_b_ based on the frequency of full‐ and half‐sibling relationships present among sampled offspring (Wang, 2009).

Another parameter that can be quantified from genetically determined pedigrees is the asymptotic number of spawning adults. The number of unique parental genotypes present in a reconstructed pedigree provides an estimate of the minimum number of spawning adults that produced the sampled offspring (*N*_s_). Estimates can then be extrapolated to the number of successfully spawning adults, minimizing limitations associated with sample size (provided that the sample is representative of the entire population), by estimating the asymptote of the pedigree accumulation curve of unique parental genotypes (Israel & May, 2010; Rawding et al., 2014). Similar to a species accumulation curve in community ecology (Chao, 1987), unique parental genotypes are accumulated as the number of sampled offspring increases. Thus, methods more commonly applied to estimate total species richness from ecological datasets (e.g., Chao, 1987) can be applied to estimate the total number of spawning adults in a system from reconstructed pedigrees ($\hat{N_{s}}$; Sard et al., 2021).

Advances in genotyping technology have allowed large‐scale genetic‐based population assessments with large sample sizes and genome‐wide SNP data, including for sea lamprey (Sard et al., 2020; Weise et al., 2020). Genomic technologies like restriction site associated DNA sequencing (RADseq; Baird et al., 2008) and RAD‐capture sequencing (Ali et al., 2016) allow for high‐throughput genotyping. Furthermore, a chromosome‐level and a germline genome have been assembled for sea lamprey (Smith et al., 2013, 2018) and a chromosomal‐anchored RAD‐capture SNP panel was recently developed (Sard et al., 2020), allowing for sequencing of large numbers of individuals at a targeted set of polymorphic loci. Population genetic data sets could be generated annually using these techniques, and used to quantify spawning abundance and provide additional information for monitoring and control under an adaptive management framework.

In this study, our objective was to evaluate the utility of *N*_b_ and *N*_s_ estimates in larval sea lamprey collections in 17 Great Lakes tributaries and quantify the influences of stream environmental, biotic, and sampling variables on genetic estimates of spawning adult abundance. Additionally, CMR estimates of *N*_c_ in a group of streams with annual adult trapping assessments were also included to allow for comparisons between *N*_c_ estimates and *N*_b_ and *N*_s_. Because aspects of sampling regimes vary in efficiencies and effective sampling areas covered, results from genetic‐based assessments may vary among stream reaches and across streams depending on the sampling regimens used to collect genotyped individuals (Hunter et al., 2020). Consequently, management decisions may also be affected by sampling protocols. Data developed by this project allow assessment of the utility of these genetic estimates for characterizing stock‐recruitment dynamics and evaluating the success of sea lamprey control measures targeting the adult life stage that also consider important stream and sampling covariates.

## 2 METHODS

### 2.1 Sample collection

**FIGURE 1:**
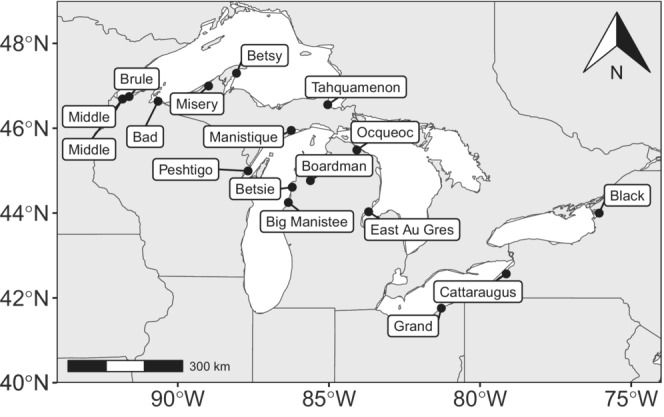
Map showing all sampled streams with their location in the Great Lakes system. Each dot represents a lake terminus of a stream system, labels indicate the name of the stream system in subsequent tables and figures.

Sea lamprey larvae (*n* = 1877) were collected by backpack electrofishing during larval assessment surveys in 17 streams across the Great Lakes basin (Figure 1) by collaborators from the United States Fish and Wildlife Service, United States Geological Survey, and Fisheries and Oceans Canada. Collections occurred at a single time for each stream in the summer and fall of 2019, with the exception of the Middle River, where collections occurred in the summer of 2017. Larval collections were made opportunistically in transects of approximately 200 m in areas of the stream where annual assessment was generally conducted. Sampled tributaries ranged from large rivers like the Muskegon River (drainage area 7327 ha) to small streams like Swan Creek (drainage area 5 ha). All systems annually recruit larval sea lamprey and received TFM treatments within 5 years of sample collection. Thirteen of the 17 streams are adult CMR index streams, although only 10 streams had a CMR abundance estimate associated with the spawning year of collected larvae. Larvae suspected to be age‐1 or age‐0 based on estimated length in the field were prioritized for collection over larger individuals in all 2019 samples. Age‐1 individuals were primarily collected and sequenced for genetic analysis (Table 1). Age‐0 individuals are only available during August and September, when larvae from early spawning adults had grown to a size that was susceptible to larval assessment surveys.

**TABLE 1:** Environmental, biotic, and sampling data for each stream.

Stream	Sample size	Sample collection		Spawning year	TFM treatment		Years since TFM treat	*N*_c_ – Spawning year	Drainage	Sample sites	Sampling distance
		Year	Month		Year	Month					
Bad	37	2019	August	2019	2017	September	2	4333 (32)	2270	1	0.2
Betsie	80	2019	May	2018	2017	June	1	1654 (*)	590	1	0.2
Betsy	123	2019	May	2018	2017	July	1	1097 (11)	230	2	4
Brule	33	2019	September	2018	2018	June	0	36,558 (13)	408	1	0.2
Cattaraugus	240	2019	April	2018	2016	May	2	1637 (70)	1129	4	11
Pigeon	51	2019	August	2019	2016	August	3	NA	1550	3	6
East Au Gres	21	2019	July	2018	2018	June	0	2124 (13)	653	2	3
Ford	122	2019	May	2018	2017	May	1	NA	1216	1	0.2
Manistique	29	2019	July	2018	2016	September	2	10,420 (4)	3631	1	0.2
Manistee	185	2019	July	2018	2016	August	2	7219 (21)	546	1	0.2
Middle	444	2017	May	2016	2013	June	3	4705*	142	7	6
Misery	37	2019	May	2018	2015	July	4	NA	102	1	0.2
Muskegon	53	2019	May	2018	2017	September	1	NA	7327	1	0.2
Ocqueoc	121	2019	May	2018	2016	July	2	4813 (5)	363	2	3
Swan	38	2019	July	2018	2013	July	5	NA	5	1	0.2
Tahquamenon	92	2019	June	2018	2015	October	3	3974 (9)	2176	1	0.2
Two‐hearted	43	2019	May	2018	2016	August	2	NA	521	1	0.2

*Note*: Treatment year and month refer to the most recent lampricide treatment that occurred in the stream, the years since treatment variable refers to the number of years between the most recent treatment and spawning year. *N*_c_ is the census‐size estimate based on mark–recapture for the years 2016 and 2018, the number in parentheses is the coefficient of variance as calculated by Adams et al., 2021, the asterix indicates that no coefficient was available (N. Johnson, personal communication). Drainage refers to the drainage area of the stream (in hectares). Sample Sites refer to the number of collection locations for the larval collections, and sampling distance is the estimated linear distance that sampling occurred in the stream (in km). For streams with one sampling site, the standard transect length for larval sampling (0.2 km) is used.

At each collection site, larvae were identified to species using morphology (Potter & Gill, 2003) and anesthetized with MS‐222, preserved in 95% ethanol, and returned to the lab for processing (IACUC Approval number: PROTO201800143). Individual lengths were recorded and a tissue sample from each larva was taken for DNA extraction and sequencing. Body length, time of sample collection, and years since TFM treatment were subsequently used to determine whether the collected individuals were age‐1 or age‐0 larvae (Table 1).

### 2.2 Sequencing library preparation

DNA extractions were performed using blood and tissue kits (Qiagen DNeasy, QIAGEN, Carlsbad, CA), following manufacturer protocols. DNA concentrations were quantified with a spectrophotometer (Nanodrop ND‐1000, ThermoFisher Scientific) and verified using assay Kits (Quant‐iT™ PicoGreen™ dsDNA, Thermo Fisher Scientific Inc.) with a real‐time PCR system (QuantStudio 6 Flex, Thermo Fisher Scientific Inc.). DNA extractions were diluted to <100 ng/μl for RAD library preparation. Samples from each stream were randomly distributed across libraries to minimize the potential for library effects.

Reduced representation libraries were constructed using a modified version of the BestRAD protocol (Ali et al., 2016). Briefly, DNA was digested with the *SbfI* restriction enzyme, and biotinylated BestRAD adapters were ligated to samples to serve as individual barcodes. The barcoded DNA was pooled, concentrated with Ampure beads (Beckman Coulter), and sheared to 325 bp using a focused‐ultrasonicator (Covaris m220, Covaris). DNA fragments with attached bestRAD tags were selected using a streptavidin bead binding assay, and size selection was used to select target size fragments of 300 bp. A 22:50 ratio of Ampure beads to sample was used to select long fragments and a 13:72 ratio was used to separate target size fragments from short fragments. Sample preparation kits (NEBNext, New England BioLabs Inc.) were used to ligate plate‐specific Illumina adaptors and an Illumina universal adapter was used to prepare the library for sequencing. Libraries were pooled in groups of four, and then a panel of ~3400 SNPs was targeted for sequencing using a custom hybridization capture kit (MyBaits, Arbor Biosciences), designed by Sard et al. (2020), with the manufacturer protocol and 11 PCR cycles in the final amplification step. Libraries were sequenced on a total of five sequencing lanes (Illumina HighSeq X) at Novogene with paired‐end 150 bp sequencing.

### 2.3 Bioinformatic analysis

A bioinformatic pipeline based on Sard et al. (2020) and Weise et al. (2020) was used to process sequencing data. First, reads were oriented with a custom perl script bRAD_flip (originally developed by Paul Hohenlohe, University of Idaho, and modified by Brian Hand and Seth Smith, University of Montana) and demultiplexed using the Stacks 2.0 (Catchen et al., 2013) function process_radtags. Cloned reads were removed from each individual with the Stacks 2.0 function cloneFilter (Catchen et al., 2013), and reads were trimmed and quality filtered with Trimmomatic (Bolger et al., 2014) with a minimum length of 50, a sliding window of four bases, and a minimum quality score of 15. BWA‐mem (Li, 2013; Li & Durbin, 2010) was then used to map all reads to the sea lamprey chromosome‐level reference genome (Timoshevskaya et al., 2023). SAMtools (version 1.9; Li et al., 2009) was used to sort mapped reads. The sorted reads were genotyped using the Stacks function gStacks (Catchen et al., 2013), and a sorted VCF file was generated along with population‐level statistics using the Stacks function populations. SNP data were initially filtered to a minimum depth of 8× for final genotype calls. For each population, HDplot (McKinney et al., 2017) was used to filter potentially paralogous loci from the data set. If the observed heterozygosity was greater than 0.6 or the absolute value of the read ratio deviation statistic (D) was greater than 7, the locus was removed from the data set (McKinney et al., 2017). Additionally, SNPs were checked for deviance from Hardy–Weinberg equilibrium across populations using the output from populations (Catchen et al., 2013). No SNPs were found to deviate from Hardy–Weinberg equilibrium in all sampled populations.

Methods for generating *N*_b_ estimates and reconstructed pedigrees have different data requirements to run optimally. Thus, two SNP sets were generated for each population using different filtering parameters: one for linkage disequilibrium (LD; Waples & Do, 2008) *N*_b_ estimates, and the other to generate a reconstructed pedigree (Wang, 2009). For both datasets, SNPs were filtered to exclude loci that were not targeted by the RAD‐capture bait panel, and loci where fewer than 80% of individuals were genotyped. The linkage disequilibrium dataset was limited to one SNP per RAD‐capture tag, where the selected SNP had the highest percentage of individuals genotyped among the SNPs on the tag with a minimum minor allele frequency (MAF) of 0.05. SNP loci in the dataset used for pedigree reconstruction in Colony (Jones & Wang, 2010) were selected using a sliding window of 1 MB to minimize physical linkage among SNPs and maintain locus independence, with selection biased toward SNPs with high MAF and high percent genotyped to maximize information content of the dataset.

### 2.4 *N*_b_ and *N*_s_ estimates

For all cohorts, two estimates of *N*_b_ along with *N*_s_ were generated. Estimates from the linkage disequilibrium method (Waples & Do, 2008) were calculated using NeEstimator (Do et al., 2013). A *p*_crit_ value of .05 was specified to exclude loci with low minor allele frequency and locus pairs within chromosomes were excluded from the calculation of correlation in allele frequency to avoid the effects of physical linkage (Waples, 2016a). Confidence intervals were estimated in NeEstimator using the provided jackknife method. Colony version 2.0.6.6 (Jones & Wang, 2010) was run for each stream population to reconstruct the pedigrees of each system and generate an estimate of *N*_b_ using the sibship frequency method. The full‐likelihood approach with a medium‐length run was used for all streams. Other input parameters changed from default settings were unknown allele frequencies, polygamous mating, and no sibship scaling or prior sibship reported (Wang & Santure, 2009). Additionally, the mean ($\bar{k}$) and variance (*V*_*k*_) of adult reproductive success for contributing adults were calculated for each reconstructed pedigree from each stream. The number of unique parental genotypes was recorded from the reconstructed pedigree (*N*_s_) and the total number of parents in the stream ($\hat{N_{s}}$) was estimated using pedigree accumulation analysis as described by Sard et al. (2021). The number of unique parental genotypes in the reconstructed pedigree was quantified as the offspring sample size increased and extrapolated using the Chao estimator (Chao, 1987) with the function specpool from the R package vegan (Oksanen et al., 2019).

### 2.5 Statistical analyses

Aspects of the sampling regime, biotic factors, and the physical environment may all influence estimates of *N*_b_ and $\hat{N_{s}}$ in the sampled systems. For instance, if larval sample sizes are small, or if sampling is not representative of the genotype diversity in the stream, *N*_b_ and $\hat{N_{s}}$ estimates may be biased. Linear models were used to assess the influence of several factors on *N*_b_, $\hat{N_{s}}$, and *V*_*k*_ for the sampled systems. Publicly available reports on current sea lamprey control and assessment were used to collect information on TFM treatment years (Barber & Steeves, 2019; Mullett & Sullivan, 2017; Steeves & Barber, 2020). Personal communications with collaborators and unpublished data from co‐authors were used to obtain data on the drainage area for each stream (J. Adams, U.S. Geological Survey, personal communications, September 2020). Input variables used for the generalized linear models are summarized in Table 1.

Generalized linear models with the above environmental variables as independent variables were generated for estimates of *N*_b_ (both LD and SF methods), $\hat{N_{s}}$, and *V*_*k*_. Predictor variables were evaluated for collinearity using variance inflation factors, calculated with the VIF function from the R package car (Fox & Weisberg, 2019). Model selection was conducted using Akaike Information Criteria (Akaike, 1974) with a correction applied for a small sample size (Burnham & Anderson, 2002), calculated with the dredge function in the MuMIn R package (Barton, 2022). Models with ΔAICc < 2 were included in the confidence set for each predictor, and coefficients were averaged across these models using the model.avg function, and are evaluated based on the results of the dredge function.

Relationships between CMR estimates of adult census size (*N*_c_; Barber & Steeves, 2019; Mullett & Sullivan, 2017; Steeves & Barber, 2020) and estimates of *N*_b_ and *N*_s_ across streams were evaluated via Pearson product–moment correlation tests. Correlations were assessed with all possible data points (*N* = 10 streams), and again after removing streams with small sample sizes (*n* < 50; *N* = 7 streams). This additional correlation for streams with large sample size was generated after sample size was found to be an important predictor of both *N*_b_ and *N*_s_ estimates in the linear models described above.

## 3 RESULTS

### 3.1 Bioinformatic processing

Larval sea lamprey was sequenced in 20 libraries in five sequencing lanes, producing an average of 234,404,375 reads per library and 2,085,919 reads per individual The average read depth per individual was 26× across all SNPs. After applying a depth filter of 8× coverage, 200,190 SNPs remained in the data set. 64.48% of identified SNPs occurred in baited loci, and an average of 16.1% of individuals were genotyped per SNP within the baited loci. The SNP sets for the linkage disequilibrium estimates contained between 2659 and 3018 SNPs with an average 94.6% percent of individuals genotyped and 0.14 average MAF. The SNP sets for pedigree reconstruction contained between 624 and 700 SNPs with 95.0% of individuals genotyped and 0.24 average MAF. Based on simulations conducted by Sard et al. (2020), sea lamprey pedigrees estimated in Colony with over 500 SNPs should be sufficient to accurately estimate full‐ and half‐sibling relationships given our expected *N*_b_ range. Between populations, there was an average of 48.5% overlap between SNPs selected for the Colony sets, and 25.8% overlap among the NeEstimator SNPs. However, there was 96.5% overlap among RAD tags for NeEstimator selected SNPs.

### 3.2 *N*_b_, *N*s, and $\hat{N_{s}}$ estimates

**FIGURE 2:**
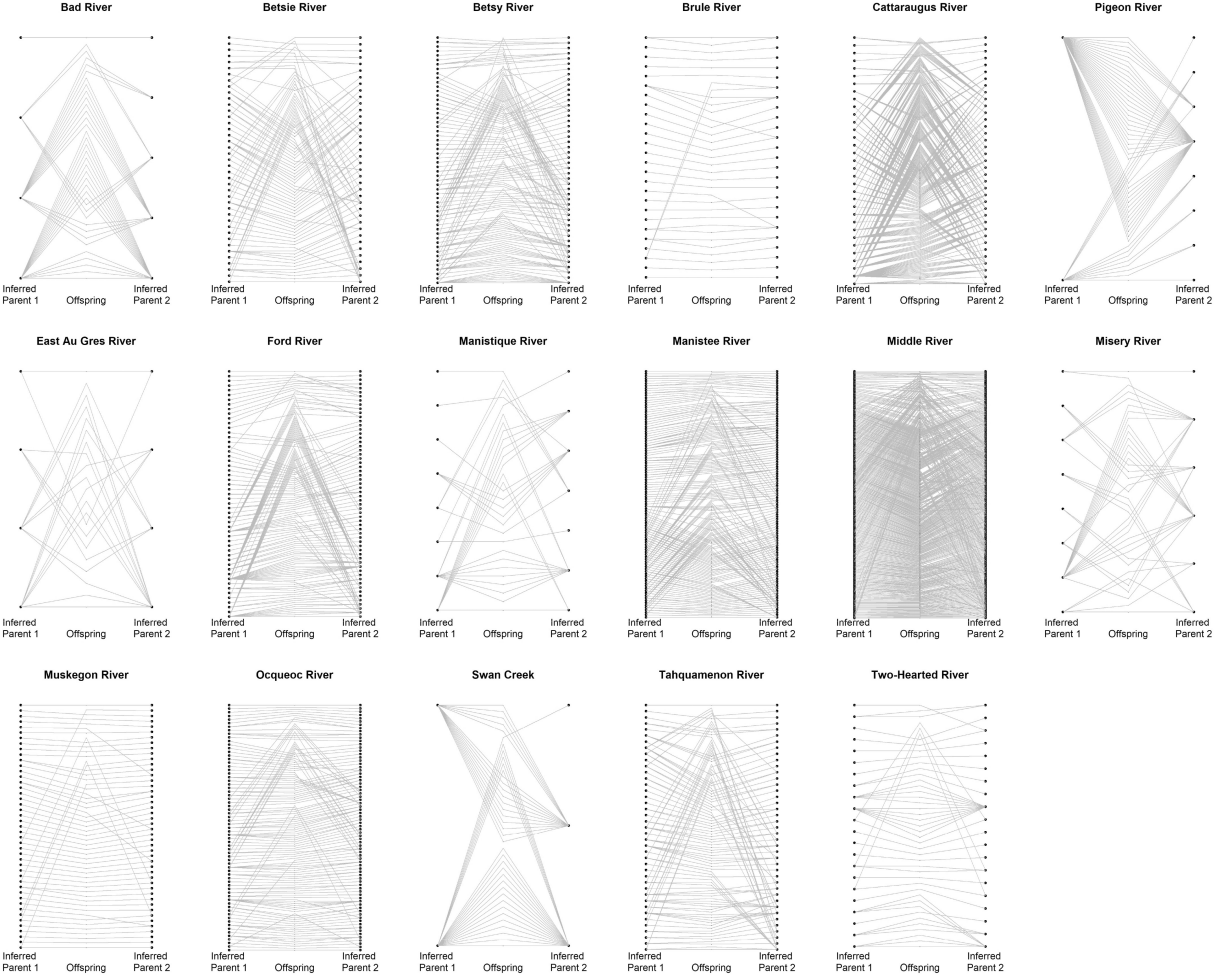
Diagrams of reconstructed larval sea lamprey pedigrees for all stream systems indicating the contrast in inferred spawning adult numbers and mating complexity. Larvae are arranged along the center of each stream diagram and are connected to their reconstructed parents indicated by dots with gray lines. The offspring are sorted by parent 1 sibling groups, then parent 2 sibling groups.

The number of families and the size of half‐sibling networks varied widely among streams, implying large differences in spawning population size and variance in individual reproductive success across streams. For example, Swan Creek and Bad River had a small number of full‐sibling families, and comparatively few half‐sibling families (Figure 2). Locations like the Ocqueoc River and the Muskegon River were represented by mostly unrelated individuals (Figure 2), while the Middle River pedigree was composed of many small, interconnected half‐sibling families. Contrastingly, Misery River was characterized by a comparatively smaller number of highly inter‐connected half‐sibling families (Figure 2).

In most systems, *N*_b_ estimates from LD and SF were of similar magnitude (Table 2). In systems where the LD method did not agree with the SF method, or when the confidence intervals did not overlap, LD estimates were generally lower (Table 2). Estimates of *N*_b_ varied widely across the sampled cohorts. The largest estimates occurred in the Middle River (*N*_b_ = 228–353) and the Muskegon River (*N*_b_ = 249–367), while six streams produced at least one *N*_b_ estimate under 10 (Table 2). Variance in individual reproductive success (*V*_*k*_) was less than 50 across most systems (mean *V*_*k*_ = 22.1, range = 0.1–177.8; Table 2), with the exception of Pigeon River (*V*_*k*_ = 177.8) and Swan Creek (*V*_*k*_ = 56.6). Mean individual reproductive success was less than 10 across the majority of streams (mean $\bar{k}$ = 4.4, range = 1.2–15.2; Table 2), with higher $\bar{k}$ in Pigeon River ($\bar{k}$ = 15.2) and Swan Creek ($\bar{k}$ = 10.2). Confidence intervals for the LD method were potentially artificially narrow due to the large number of SNPs used in the analyses (Waples et al., 2022), although the corrected jackknife estimates should reduce that bias. All seven streams with small sample size (*n* < 50) had LD *N*_b_ estimates below 100. Of these locations, two had mark–recapture estimates of over 10,000 and *N*_s_ estimates of less than 100 (Table 2).

**TABLE 2:** *N*_b_ and *N*_s_ estimates and population‐based information.

Stream	Sample size	*N*_b_ – LD	*N*_b_ – SF	*N*_s_	Chao	$\bar{k}$	*V*_*k*_
Bad	37	3 (2–3)	6 (3–15)	9	10 ± 2	8.2	37.1
Betsie	80	62 (48–82)	80 (55–116)	79	97 ± 48	2.0	1.9
Betsy	123	57 (46–73)	80 (59–108)	104	165 ± 46	2.4	4.0
Brule	33	68 (37–218)	111 (68–208)	51	161 ± 37	1.3	0.4
Cattaraugus	240	33 (29–37)	40 (27–65)	70	76 ± 29	6.9	42.4
Pigeon	51	7 (6–9)	4 (2–12)	10	11 ± 6	10.2	177.8
East Au Gres	21	0.2 (0.2–0.3)	7 (3–21)	8	8 ± 0.2	5.3	7.2
Ford	122	38 (29–52)	40 (26–63)	113	182 ± 29	2.2	10.5
Manistique	29	7 (4–10)	11 (6–27)	15	27 ± 4	3.9	8.1
Manistee	185	144 (117–184)	207 (167–262)	207	440 ± 117	1.8	1.8
Middle	444	228 (202–259)	353 (302–420)	402	545 ± 202	2.2	2.9
Misery	37	9 (7–11)	9 (5–24)	14	14 ± 7	5.3	17.6
Muskegon	53	249 (172–428)	367 (237–764)	91	279 ± 172	1.2	0.1
Ocqueoc	121	133 (102–182)	180 (140–239)	145	239 ± 27	1.7	1.1
Swan	38	2 (2–2)	4 (2–12)	5	5 ± 2	15.2	56.6
Tahquamenon	92	50 (38–70)	68 (47–70)	82	121 ± 38	2.2	3.2
Two‐hearted	43	25 (17–40)	30 (18–51)	42	77 ± 20	2.0	3.6

*Note*: Sample size indicates the number of sequenced offspring for the cohort. Linkage disequilibrium (LD) and sibship frequency (SF) columns provide effective breeding size (*N*_b_) point estimates and corresponding 95% confidence intervals. $\bar{k}$ and *V*_*k*_ are the mean and variance in reproductive success of contributing parents, as inferred from the reconstructed pedigree. *N*_s_ is the number of reconstructed parental genotypes for each cohort, and Chao is the $\hat{N_{s}}$ point estimate for the extrapolated number of spawning adults ($\hat{N_{s}}$) and 95% confidence interval.

**FIGURE 3:**
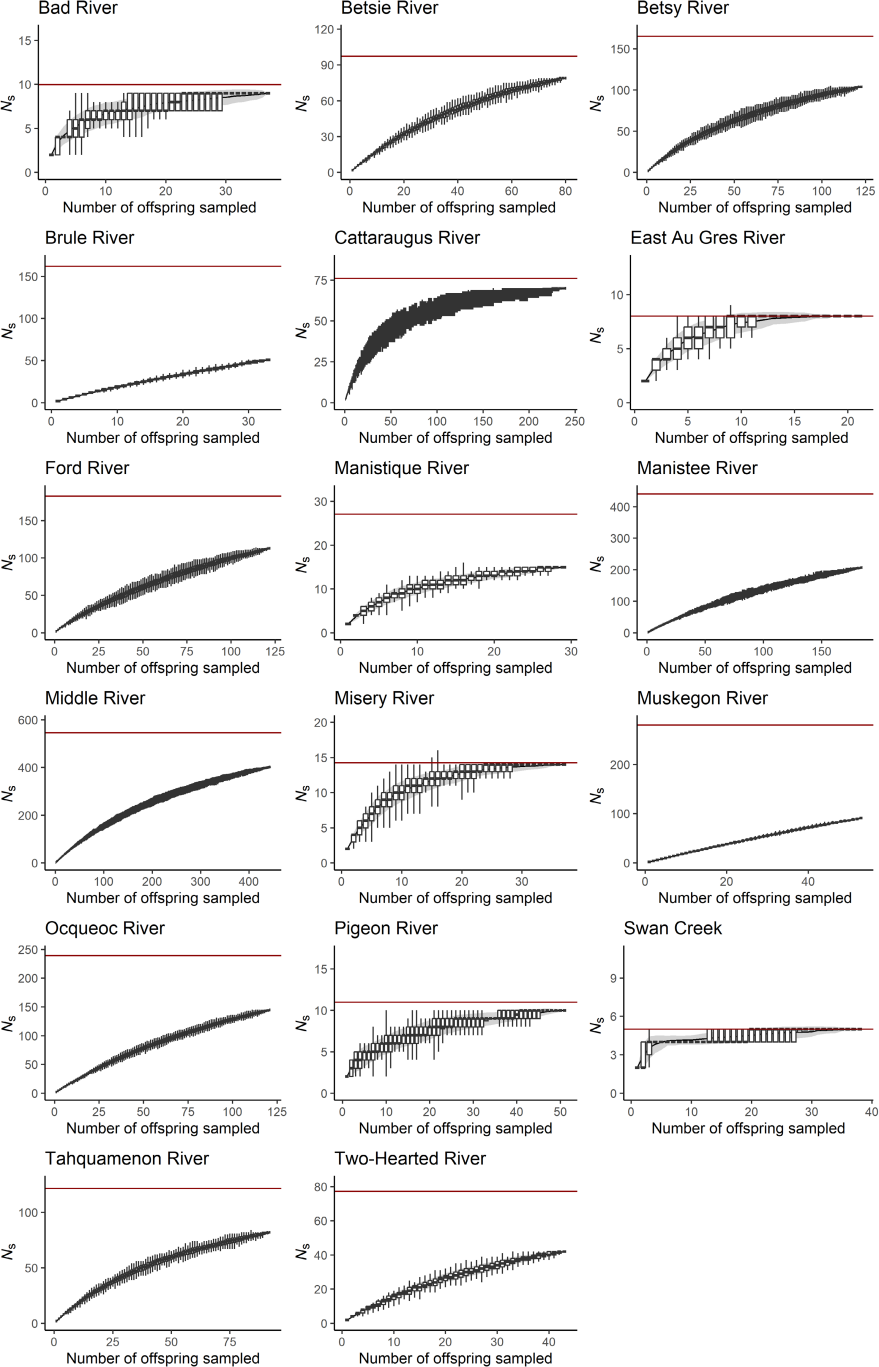
Pedigree accumulation curves (Sard et al., 2021) describing increases in numbers of unique parent genotypes inferred (*N*_s_) as a function of increases in the number of sampled offspring accumulated in each stream. Estimates of $\hat{N_{s}}$ are indicated by the dark red line on each plot.

Estimates of $\hat{N_{s}}$ were generally higher than the *N*_b_ estimates for each cohort. Some systems had a small sample size (less than 50 individuals), but finite $\hat{N_{s}}$ estimates were calculable due to the fact that all stream subpopulations contained some related individuals (Table 2). The Bad River and the East Au Gres River produced small $\hat{N_{s}}$ estimates where accumulation curves reached their asymptotes within the sample (Figure 3). In contrast, $\hat{N_{s}}$ estimates were larger in the Manistee and Middle Rivers, and an asymptote in the relationship between sampled offspring and the number of unique parental genotypes was not reached (Figure 3).

### 3.3 Correlations and linear modeling

**FIGURE 4:**
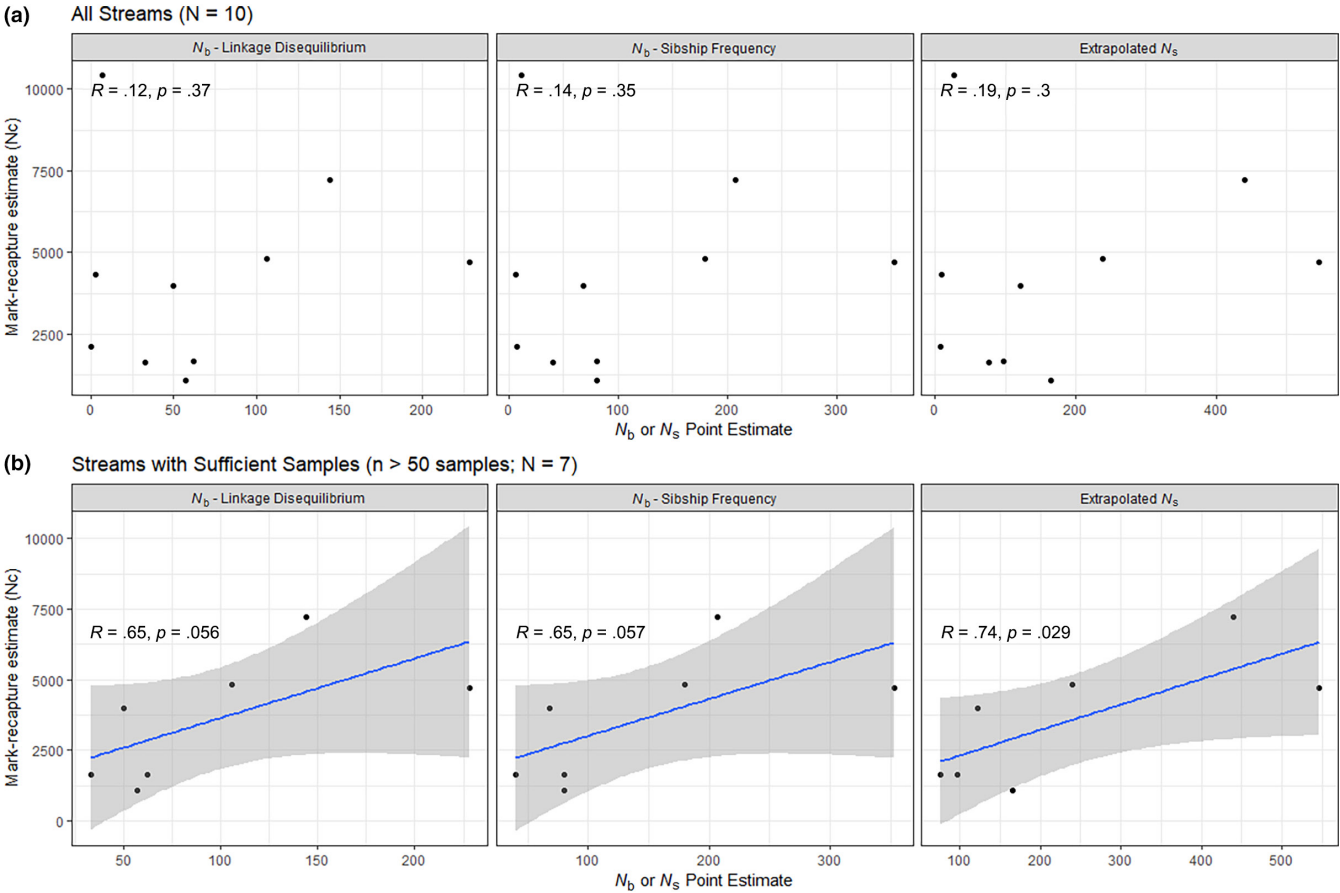
Evaluation of relationships between effective breeding size (*N*_b_) and minimum number of spawning adults (*N*_s_) estimates and mark–recapture census size estimates (*N*_c_) estimates for sequenced stream populations. Correlation coefficients (*R*) and *p*‐values are listed in each subplot, relationship is shown with a blue line and a gray outline for the uncertainty. Due to low correlation coefficients, relationships are not visualized for the top row of subplots.

A correlative relationship between stream estimates of *N*_c_ and *N*_b_ or $\hat{N_{s}}$ was not evident when data from all sampled locations with mark–recapture census size estimates were considered (Figure 4a). However, when streams with small sample size (*n* < 50) were removed from the analysis, correlation coefficients for the relationship between *N*_c_ and all three genetic estimates increased (*R* = .65–.74), indicating that small sample sizes may have introduced a bias in genetic estimates (Figure 4b). *N*_b_ and $\hat{N_{s}}$ estimates were positively correlated (SF‐based *N*_b_ and $\hat{N_{s}}$: *R* = .954; LD‐based *N*_b_ and $\hat{N_{s}}$: *R* = .951).

The VIF analysis found collinearity among the full set of environmental, biotic, and sampling variables that could be considered in the models. When variables were subset to sample size, sampling distance, years since TFM treatment, and drainage area, VIF results decreased to acceptable levels (GVIF < 2), indicating that collinear variables were successfully removed. The variables that were removed were the month of TFM treatment, which was correlated with years since TFM Treatment, and the number of sampling sites, which was correlated with sampling distance.

**FIGURE 5:**
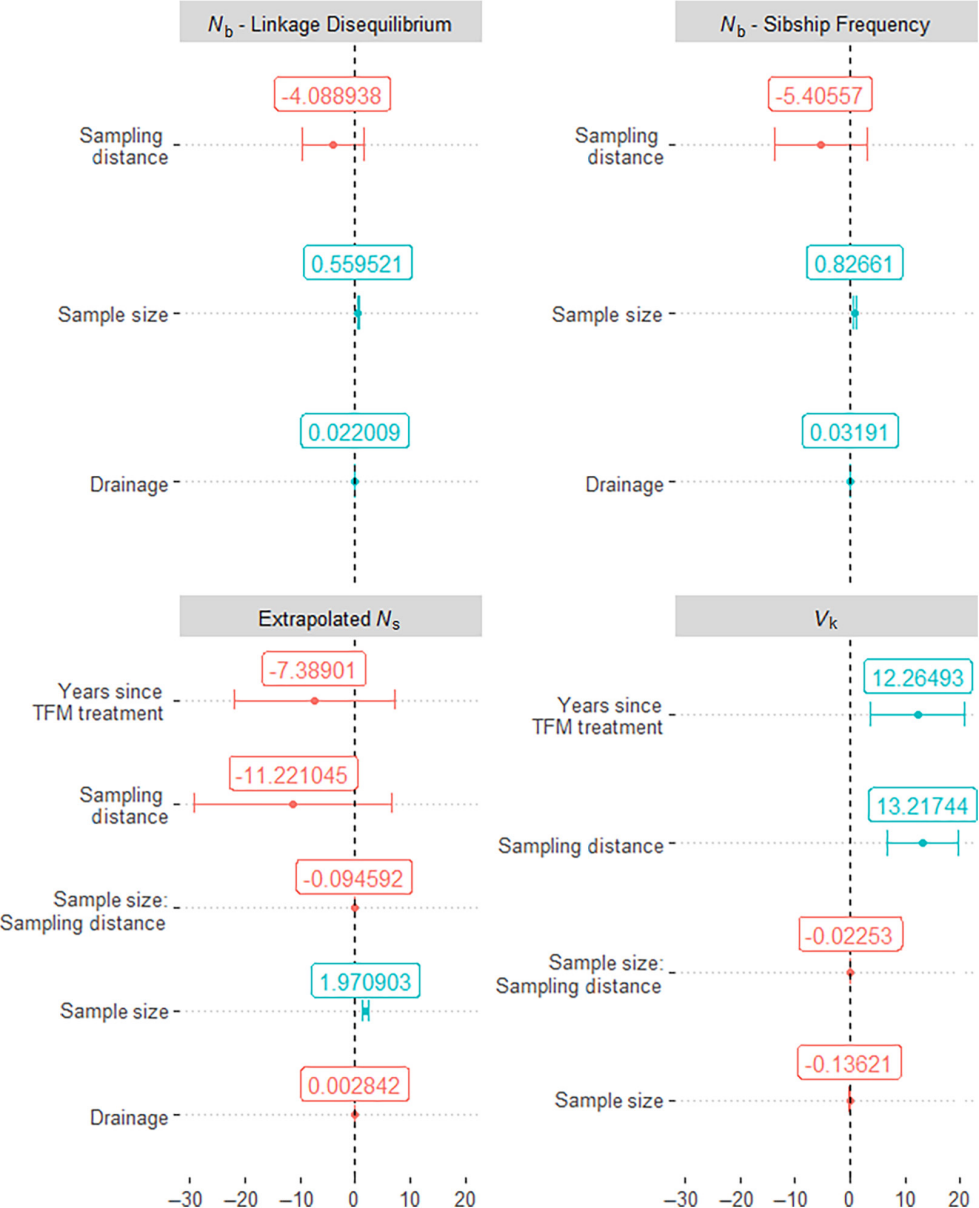
Visualization of model‐averaged coefficients describing environmental and response variables for generalized linear models generated for four different genetic estimates. Coefficients with no error bar overlap with zero are shown in blue, coefficients with overlap are shown in red. Zero is plotted with a black dotted line on each subplot.

Sample size and sampling distance were found to be important predictors in the confidence set of models for *N*_b_, $\hat{N_{s}}$, and *V*_*k*_ estimates (Table 3). Drainage area was an important predictor in both *N*_b_ and $\hat{N_{s}}$ models, and years since TFM Treatment was an important predictor in the *N*_s_ and *V*_*k*_ model (Table 3). Models incorporating an interaction between sample size and sampling distance were also included in the confidence set for $\hat{N_{s}}$ and *V*_*k*_ with a negative effect (Table 3). However, in the $\hat{N_{s}}$ and *V*_*k*_ models, the model‐averaged coefficients for several predictors had confidence intervals that overlapped zero, indicating that there is uncertainty in the directional effect of those predictors (Figure 5). In the *N*_b_ models, confidence intervals for sampling distance overlapped zero, but coefficients for sample size and drainage area suggest positive effects on *N*_b_ estimates for both predictors (Figure 5).

**TABLE 3:** Results from environmental, biotic, and sampling linear models.

Model			
LD – *N*_b_ ~ drainage + Sample size + Sampling distance	df	AICc	ΔAICc
LD – *N*_b_ ~ Drainage + Sample size	4	190.930	0.000
LD – *N*_b_ ~ Drainage + Sample size + Sampling distance	5	191.176	0.025
SF – *N*_b_ ~ Drainage + Sample size + Sampling distance	df	AICc	ΔAICc
SF – *N*_b_ ~ Drainage + Sample size	4	206.477	0.000
SF – *N*_b_ ~ Drainage + Sample size + Sampling distance	5	207.165	0.689
Chao ~ Drainage + Sample size + Sampling distance + Sample size: Sampling distance + Years since TFM treatment	df	AICc	ΔAICc
Chao ~ Sample size + Sampling distance + Sample size: Sampling distance	5	207.823	0.000
Chao ~ Sample size + Sampling distance	4	208.136	0.313
Chao ~ Sample size + Sampling distance + Years since TFM treatment	5	208.854	1.031
Chao ~ Drainage + Sample size + Sampling distance + Sample size: Sampling distance	6	209.082	1.258
Chao ~ Drainage + Sample size + Sampling distance + Years since TFM treatment + Sample size: Sampling distance	6	209.612	1.789
*V*_*k*_ ~ Years since TFM treatment + Sample size + Sampling distance + Sample size: Sampling distance	df	AICc	ΔAICc
*V*_*k*_ ~ Sample size + Sampling distance + Years since TFM treatment	5	178.148	0.000
*V*_*k*_ ~ Sample size + Sampling distance + Years since TFM treatment + Sample size: Sampling distance	6	178.506	0.358
*V*_*k*_ ~ Sample size + Sampling distance	4	179.791	1.643

*Note*: Model in bold indicates the averaged model, and the other models included are models selected by an AICc cutoff of 2 to be included in the average. The “+” indicates a parameter included in the model, and “a” indicates an interaction between parameters.

Abbreviations: AICc, Akaike Information Criterion; Chao, Estimate of minimum number of spawning adults extrapolated using the Chao Method; df, degrees of freedom; LD – *N*_b_, Estimate of effective breeding size using the linkage disequilibrium method; SF – *N*_b_, Estimate of effective breeding size using the sibship frequency method; *V*_*k*_, Variance in reproductive success.

## 4 DISCUSSION

Genetic assessment of 17 sea lamprey‐producing streams provided information pertaining to sea lamprey reproductive ecology, including mean and variance in reproductive success and full‐ and half‐sibling family size, that could not be obtained from other types of adult assessment. Variability in estimates of *N*_b_ and *N*_s_ among streams was notable, indicating that sea lamprey‐producing streams have vastly different numbers of successfully spawning adults (Table 2). The number and size of full‐ and half‐sibling families in each stream varied widely, further illustrating variation among Great Lakes tributaries and highlighting the importance of evaluating lamprey spawning populations on a per‐stream basis to inform management decisions. Patterns of family structure ranged from cohorts comprised of a small number of full‐sibling families, to those consisting of mostly unrelated individuals, to cohorts with large, interconnected half‐sibling groups (Figure 2). Variation in the number of spawning adults and their subsequent spawning success may be driven partially by the sea lamprey's lack of homing behavior (Bergstedt & Seelye, 1995; Waldman et al., 2008) and dependence on larval olfactory cues during adult spawning migrations (Wagner et al., 2009). Sample size and the sampling distance were identified as influential predictors of *N*_b_ and $\hat{N_{s}}$in the confidence set of models, along with physical stream features and past stream management regimes, emphasizing the importance of representative sampling. While *N*_b_ and $\hat{N_{s}}$ were not significantly correlated with adult census size in the full dataset, relationships were marginally insignificant for well‐sampled tributaries (Figure 4b), suggesting that these population genetic parameters could serve as useful surrogates for spawning adult census size. Thus, population genomic datasets like those generated here provide a promising approach for population assessment, particularly in systems where adult trapping is difficult or impossible. However, the accuracy of *N*_b_ and $\hat{N_{s}}$estimates depends critically on experimental design.

### 4.1 Sampling schemes and generalized linear models

Across the streams surveyed, the importance of sampling design, particularly sample size, was highlighted in findings from generalized linear models and correlations between *N*_b_ and *N*_c_ CMR estimates. When streams with small sample size were removed from the dataset, larger correlation coefficients were estimated between *N*_b_ and $\hat{N_{s}}$ estimates and *N*_c_ (Figure 4). Collectively, results indicate that the opportunistic sampling scheme used to collect sea lamprey larvae in this study, which produced small sample sizes in several streams, may have led to inaccurate representations of larval family composition in some streams. Beyond the sampling variables noted here, there were also environmental predictors with effects on genetic estimates that were identified as important in our linear models when all streams were included in models, as discussed below.

The relationship between *N*_b_ and *N*_s_ in sampled Great Lakes streams was shown to be associated with elements of the sampling regime (larval numbers collected, number of sampling sites, and lengths of stream surveyed), stream and environmental factors (drainage area), and management control efforts (recency of TFM treatment). Previous work estimating *N*_c_ in sea lamprey considered variables similar to the environmental data used in the generalized linear models in this study (Mullett et al., 2003). These variables included drainage area and years since the last TFM treatment, which were included in some of the best models describing associations with *N*_b_ and *N*_s_ estimates (Table 3). In the *N*_b_ models, drainage area and sample size had a positive effect on both estimates of *N*_b_, with similar effect sizes, indicating that increasing sample size and larger drainage area lead to larger *N*_b_ in our sampled streams. Sampling distance had a negative effect on *N*_b_ estimates, implying that increasing the distance sampled decreased *N*_b_ estimates in our sampled streams, which conflicts with our expectations. However, both coefficients have confidence intervals that overlap zero, implying some uncertainty in the direction of the effect. Years since TFM treatment had a negative effect on $\hat{N_{s}}$ but a positive effect on *V*_*k*_, implying that a recent TFM treatment would lead to less variation in reproductive success and a larger number of unique parent genotypes, the latter of which conflicts with expectations due to the fact that a recent TFM treatment should decrease larval cue and thus the number of adults that enter a stream to spawn (Mullett et al., 2003). The coefficient for the $\hat{N_{s}}$ model did have confidence intervals that overlapped zero, however.

Given variation in sample sizes across the streams in our study, and the consistent inclusion of sampling attributes in linear models relating *N*_b_ and $\hat{N_{s}}$ to stream‐specific covariates, it seems likely that stream cohorts characterized based on smaller sample sizes did not adequately characterize the spawning populations that produced the sampled cohorts. Sources of uncertainty with *N*_c_ estimates could have further complicated correlations involving those estimates. Low trap efficiency and variation in trap efficiency across years and index streams, as well as variation in catchability for individual lamprey could contribute to uncertainty in *N*_c_ (Harper et al., 2018). In two of our streams, *N*_c_ estimates were greater than 10,000, while the number of sampled offspring was less than 50 (Table 1), and the sample was collected from a single site in the stream. In these systems, non‐random sampling may have produced a downward bias in *N*_b_ and *N*_s_ estimates. Additionally, if the system had high variance in reproductive success, the outsized effect of a few parents would decrease the *N*_b_ : *N*_c_ ratio (Hedrick, 2005). We found substantial variation in V_k_ among streams (Table 2), which may obscure correlations between our *N*_b_ estimates and *N*_c_ estimates from CMR efforts. Tests for correlations between *N*_b_, $\hat{N_{s}},$ and *N*_c_ were conducted with and without locations with small samples and the relationships between *N*_c_ and genetic estimates were strengthened when these systems were removed (Figure 4). When sample size and sampling distance were small, non‐representative sampling may have resulted in downwardly biased *N*_b_ and $\hat{N_{s}}$ estimates (Waples & Anderson, 2017). This same bias applies when a small sample size is used for CMR estimates, compounding differences between genetic and CMR assessment. To reduce such bias, sampling would be best conducted at multiple stream locations that are distributed across the available larval habitat. We further assessed the influences of the sampling scheme in the Middle River, where three sampling sites had sufficient collections for an *N*_b_ estimate (*n* > 30 individuals). We generated site‐specific *N*_b_ estimates for all three locations using the LD estimation methods and found that, while point estimates for individual sites ranged from 292 to 394, all three locations produced estimates with confidence intervals that included the estimate from the combined sample. Thus, our results would not have been markedly different for the Middle River, had our estimates been based on a more spatially restricted set of samples.

### 4.2 Relationship between *N*_b_ and *N*_c_

In previous studies, population estimates of *N*_b_ and *N*_c_ have not always exhibited the expected correlations (Bernos & Fraser, 2016; Whiteley et al., 2015) especially when the population size (*N*_c_) was large (Waples, 2016b). However, some studies have found a relationship when environmental factors and population dynamics, and their influences on the *N* : *N*_c_ ratio, could be taken into account (Ruzzante et al., 2016). In particular, the importance of sufficient and representative sampling has been highlighted (Whiteley et al., 2012), which is consistent with results from our study (Table 3; Figure 4). Additional environmental variables not included in our models, including the amount and distribution of spawning habitat relative to sampling effort, density of spawning adults, and stream flow during spawning could also affect *N*_b_ and *N*_s_ (Whiteley et al., 2015). A further potential complication is genetic compensation, when variation in reproductive success decreases in small populations, inflating *N*_b_ estimates compared to *N*_c_ (Ardren & Kapuscinski, 2003; Whiteley et al., 2015). The increase in correlation between *N*_b_ and *N*_c_ estimates after removing small sample size streams in this study, and the positive relationships between factors associated with sampling regimes and *N*_b_ estimates in our models, underscores the need for large and representative sampling when estimating *N*_b_ from population genomic data.

### 4.3 Implications for management

Our study illustrates the potential of *N*_b_ and $\hat{N_{s}}$ estimates as sources of information about sea lamprey spawning ecology in Great Lakes tributaries. Population genomic approaches provide tools for the annual assessment of sea lamprey spawning populations, which would be particularly beneficial in lamprey‐producing streams where adult trapping is difficult or impossible. Furthermore, if index streams are to be assessed over a large number of years, families and cohorts can be tracked through time using pedigree reconstruction, providing insights on larval growth, dispersal, and survival in streams (Lewandoski et al., 2021). *N*_b_ and $\hat{N_{s}}$ could also be used to evaluate the efficacy of supplemental control techniques including sterile male release and applications of chemical repellant/attractants to increase trapping efficiency or as barriers to upstream migration (Miehls et al., 2021; Siefkes et al., 2021). These new control techniques are being evaluated for use in streams where TFM treatment and barriers are difficult to use, and as supplements for these techniques where the negative ecological effects of physical barriers and lampricides are high (Siefkes et al., 2021). Additionally, *N*_b_ provides information on inbreeding, drift, and loss of diversity in the population, all of which can be used to further evaluate current and future control techniques. $\hat{N_{s}}$ estimates also can provide an annual metric of the number of successfully spawning lamprey in a stream, which could be used as an assessment metric complementary to *N*_c_. More in‐depth assessment of lamprey spawning provides insights into successfully reproducing adult lamprey that can be incorporated into adaptive management frameworks to make more precise intervention decisions to reduce lamprey spawning populations.

Our study identified associations between widely used genetic pedigree‐based parameters that are surrogates of mark–recapture spawning adult census size estimates and management and sampling regimes and stream physical features. Future genetic work with stratified or strategic larval sampling could provide additional insight into the relationship between *N*_b_, $\hat{N_{s}}$, and *N*_c_, and clarity on the environmental factors that influence sea lamprey spawning abundance in Great Lakes tributaries. Additionally, by pairing annual evaluation of *N*_b_ and $\hat{N_{s}}$ with multi‐year pedigree reconstruction (Bergstedt & Seelye, 1995; Waldman et al., 2008), future studies can both quantitatively assess control effectiveness and improve our understanding of the larval phase of the sea lamprey life cycle.

## AUTHOR CONTRIBUTIONS

**Ellen M. Weise:** Conceptualization (equal); formal analysis (lead); investigation (equal); methodology (lead); validation (equal); visualization (lead); writing – original draft (lead); writing – review and editing (lead). **Kim T. Scribner:** Conceptualization (equal); funding acquisition (equal); methodology (equal); supervision (equal); writing – review and editing (equal). **Olivia Boeberitz:** Formal analysis (equal); methodology (equal); writing – review and editing (equal). **Gale Bravener:** Funding acquisition (equal); project administration (equal); writing – review and editing (supporting). **Nicholas S. Johnson:** Conceptualization (equal); funding acquisition (equal); project administration (equal); writing – review and editing (equal). **John D. Robinson:** Conceptualization (equal); funding acquisition (equal); supervision (equal); writing – review and editing (lead).

## FUNDING INFORMATION

We thank the Great Lakes Fishery Commission (GLFC) for their funding used for the project (2019_ROB_540840).

## BENEFIT‐SHARING STATEMENT

We plan to work with USGS, USFWS, and DFO collaborators to share and disseminate work for sea lamprey management, and methods presented in the paper will be used as an assessment framework for future management projects.

## Data Availability

Sequencing read data will be uploaded to NBCI SRA upon acceptance of manuscript. Length data and environmental variable data are available on GitHub (https://github.com/weiseell/NbdLamprey2).
